# Mouse Nudt13 is a Mitochondrial Nudix Hydrolase with NAD(P)H Pyrophosphohydrolase Activity

**DOI:** 10.1007/s10930-017-9734-x

**Published:** 2017-07-28

**Authors:** Salama R. Abdelraheim, David G. Spiller, Alexander G. McLennan

**Affiliations:** 1 Department of Biochemistry, Institute of Integrative Biology, University of Liverpool, Liverpool, L69 7ZB UK; 2 Department of Biochemistry, Faculty of Medicine, Minia University, Minia, 61519 Egypt; 3 Faculty of Biology, Medicine and Health, Systems Microscopy Centre, University of Manchester, Manchester, M13 9PT UK

**Keywords:** Nudix, NADH, Mitochondria, Nucleotide metabolism, Pyrophosphatase

## Abstract

The mammalian NUDT13 protein possesses a sequence motif characteristic of the NADH pyrophosphohydrolase subfamily of Nudix hydrolases. Due to the persistent insolubility of the recombinant product expressed in *Escherichia coli*, active mouse Nudt13 was expressed in insect cells from a baculovirus vector as a histidine-tagged recombinant protein. In vitro, it efficiently hydrolysed NADH to NMNH and AMP and NADPH to NMNH and 2′,5′-ADP and had a marked preference for the reduced pyridine nucleotides. Much lower activity was obtained with other nucleotide substrates tested. *K*_m_ and *k*_cat_ values for NADH were 0.34 mM and 7 s^−1^ respectively. Expression of Nudt13 as an N-terminal fusion to green fluorescent protein revealed that it was targeted exclusively to mitochondria by the N-terminal targeting peptide, suggesting that Nudt13 may act to regulate the concentration of mitochondrial reduced pyridine nucleotide cofactors and the NAD(P)^+^/NAD(P)H ratio in this organelle and elsewhere. Future studies of the enzymology of pyridine nucleotide metabolism in relation to energy homeostasis, redox control, free radical production and cellular integrity should consider the possible regulatory role of Nudt13.

## Introduction

Mammalian genomes typically possess 20–25 genes for members of the Nudix superfamily. Nudix proteins hydrolyze or bind a wide variety of nucleotide and other phosphorylated molecules and are involved in many processes including nucleotide pool regulation, metabolic control and RNA decapping [1, 2]. Several Nudix hydrolases have broad substrate specificities in vitro, making it difficult to ascertain their functions in vivo [2]. This uncertainty is compounded by the common misannotation of uncharacterized Nudix proteins in online databases as, for example, ADP-ribose pyrophosphatases or antimutator 8-oxo-dGTPases based on sequence similarities to well characterized proteins with these activities; thus, experimental characterization is important. Most mammalian nudix proteins have been well studied, but a few, such as NUDT13, have not. NUDT13 is annotated in some databases as a mitochondrial NADH pyrophosphohydrolase. This is based on the presence of a putative N-terminal mitochondrial targeting sequence and the sequence motif “SQPWPFPxS” that is found in all characterized NADH pyrophosphohydrolases downstream of the catalytic nudix box [3]. A mitochondrial location has also been suggested from a proteomic study [4]. Here, we experimentally confirm these predictions for the first time with recombinant mouse Nudt13 expressed in a baculovirus system. This should now allow the potential influence of Nudt13 and its orthologs to be included in studies of nicotinamide dinucleotide metabolism, energy homeostasis, mitochondrial dynamics and disease where it has hitherto not been considered.

## Materials and Methods

### Materials

RIKEN clone 3110052E14, a full-length cDNA insert from 13-day mouse embryo head cloned between the *Xho*I and *Sst*I sites of pBluescript I SK(+), was obtained from RIKEN (the Institute of Physical and Chemical Research), Yokohama, Japan. Bac-N-Blue linear viral DNA, pBlueBac4.5/V5-His vector, *Escherichia coli* TOP10, Sf21 (*Spodoptera frugiperda*) and High Five (*Trichoplusia ni*) insect cells, Sf-900 II SFM medium, Cellfectin and MitoTracker Red CM-H_2_XRos were from Invitrogen (Thermo Fisher Scientific). pEGFP-N1 and pEGFP-C2 were from Clontech. EX-CELL 405 medium was from Sigma. FuGENE was from Roche. The anti-His.Tag monoclonal antibody was from Merck.

### Cloning of Nudt13 from cDNA into Baculovirus Vector

The mouse *Nudt13* gene was PCR-amplified from clone 3110052E14 using the forward and reverse primers 5′-CAGACTCGAGAATGAATCGGACAATGTCTC-3′ and 5′-CCATTTAAGCTTAGCAGCCAGGG-3′ which provided a *Xho*I site at the start of the amplified gene and a *Hin*dIII site at end. After amplification with *Pfu* DNA polymerase, the *Nudt13* PCR product was purified using a Qiagen PCR purification kit and digested with *Xho*I and *Hin*dIII. The digest was gel-purified and the product ligated between the *Xho*I and *Hin*dIII sites of the pBlueBac4.5/V5-His vector. The resulting pBlueBac-Nudt13 construct (10 ng), encoding Nudt13 with a C-terminal His.Tag and V5 epitope under the control of the strong polyhedrin promoter, was electroporated into *E. coli* TOP10 cells for propagation and its structure confirmed by sequencing.

Recombinant Nudt13 virus was obtained by co-transfection of the pBlueBac-Nudt13 DNA construct with linearized Bac-N-Blue viral DNA in Sf21 cells. pBlueBac-Nudt13 DNA (2 µg) was mixed with 0.5 µg Bac-N-Blue DNA in 1.5 ml Sf-900 II SFM and then 20 µl of Cellfectin was added, mixed for 10 s, then incubated for 45 min at room temperature. Sf21 cells (10^6^ cells/60 mm dish) were washed with 4 ml Sf-900 II SFM and the transfection mixture added. After 4 days at 27 °C, pure recombinant Nudt13 plaques were isolated from the viral supernatant by blue/white color selection and plaque purification using Sf21 cells [5]. The structures of the recombinants were confirmed by PCR analysis of purified viral DNA and a high titer Nudt13 viral stock (5 × 10^8^ pfu/ml) prepared from purified virus [5].

### Expression and Purification of Nudt13

After optimisation of the time and multiplicity of infection (MOI) for expression of Nudt13, High Five™ cells were seeded as a monolayer in EX-CELL 405 medium in 10 × 75 mm^2^ flasks at 10^7^ cells/flask at 27 °C then infected with recombinant Nudt13 virus at a MOI of 10. After 48 h, the cells were dislodged and centrifuged at 1000×*g* for 10 min at 4 °C, then washed with PBS. The cells were lysed in 5 ml 50 mM Tris–HCl, pH 8, 50 mM NaCl, 1% (v/v) Triton X-100, 1% (v/v) Nonidet P-40, and 1 mM phenylmethylsulfonylfluoride. After 2 h at 4 °C, the lysate was sonicated four times, 20 s each time. The extract was centrifuged at 15,000×*g* for 20 min at 4 °C and the supernatant mixed with 1 ml NiCAM™-HC resin (Sigma) equilibrated in 50 mM Tris–HCl, pH 8.0, 500 mM NaCl and gently shaken for 2 h at 4 °C. The mixture was then poured into a 15 × 50 mm column, the column washed with 2 × 10 ml 50 mM Tris–HCl, pH 8.0, 500 mM NaCl, 10 mM imidazole, and the protein eluted with 3 ml 50 mM Tris–HCl pH 8.0, 500 mM NaCl, 0.25 M imidazole. The purified protein was dialysed overnight against 2 × 1 l of 50 mM Tris–HCl pH 8.0, 50 mM NaCl, 1 mM dithiothreitol.

### Nudt13-EGFP Fusion Constructs and Subcellular Localization

The same PCR product used to make the pBlueBac-Nudt13 construct was used to make N- and C-terminal fusions of Nudt13 to enhanced green fluorescent protein (EGFP). It was ligated between the *Xho*I and *Hin*dIII sites of pEGFP-N1 or pEGFP-C2 to give pNudt13-EGFP or pEGFP-Nudt13 respectively. The plasmids were propagated by transformation of *E. coli* TOP10 cells. HeLa cells, 6 × 10^4^ cells/dish, were seeded into 35 mm glass-bottomed dishes (MatTek, Ashland, MA, USA) in 2 ml complete MEM and transfected after 24 h when at 50% confluence. FuGENE (2.5 µl/µg DNA) was diluted into 100 µl serum-free MEM, incubated for 5 min at room temperature and added dropwise to 1 µg pNudt13-EGFP or pEGFP-Nudt13 in a volume of 10 µl. The mixture was incubated for 45 min at room temperature. The old medium was removed from the dishes and replaced with 2 ml of fresh complete MEM and then the transfection mixture was added dropwise to the cell monolayer. The cells were incubated for up to 24 h at 37 °C in a humidified incubator containing 5% CO_2_. Mitochondria were visualized by incubating HeLa cells 16–24 h after transfection with pNudt13-EGFP in complete MEM containing 50–100 nM of MitoTracker red for 45 min at 37 °C in a humidified incubator in 5% CO_2_. After removal of the dye, cells were observed in the confocal microscope as previously described [6].

### Enzyme Assays

The standard colorimetric assay (200 µl) for determining optimal substrate and conditions was incubated at 37 °C for 20 min and contained 50 mM Tris–HCl, pH 8.0, 2 mM MnCl_2_, 1 mM dithiothreitol, 0.5 mM substrate, 1 µg Nudt13 and 0.5 µg (1 unit) alkaline phosphatase for phosphodiester substrates or 0.5 µg (100 mU) inorganic pyrophosphatase for phosphomonoesters. Phosphate released was determined colorimetrically [7]. Reaction products were identified by high performance ion-exchange chromatography after incubation of 0.25 mM substrate with 0.25 µg Nudt13 in 100 µl 50 mM Tris–HCl, pH 8, 2 mM MnCl_2_, 1 mM dithiothreitol, at 37 °C for 10 min [8]. Kinetic constants were determined using a substrate range from 0.05 to 0.9 mM and 0.25 µg protein.

## Results

### The Nudt13 Sequence

The mouse *Nudt13* gene encodes a 356 amino acid, 39.6 kDa protein (GenBank Accession No. BAB29203) with 78% identity to human NUDT13. It comprises two structurally similar domains separated by a rubredoxin-like zinc finger (residues 164–195) [9]. The C-terminal domain (residues 196–318) has a canonical Nudix fold [10] and contains both the catalytic Nudix box and the downstream “SQPWPFPxS” motif and is 41% identical to the equivalent region of mouse Nudt12, a mouse peroxisomal NADH pyrophosphohydrolase [6] and 39% identical to the C-terminal region of *Arabidopsis thaliana* AtNUDX19 NADPH pyrophosphohydrolase [11]. The N-terminal domain (residues 46–162) has a rudimentary Nudix fold preceded by a 27-residue sequence predicted by MitoProt (97%), and TargetP (85%) to comprise a mitochondrial leader sequence [12, 13].

### Cloning, Expression and Purification of Nudt13

All attempts to obtain recombinant Nudt13 by expression in *E.coli* yielded insoluble, inactive protein, which may explain the lack of any study reporting the properties of this enzyme so far. However, we were successful with a baculovirus expression system. The *Nudt13* sequence was PCR-amplified from a full-length mouse embryo cDNA and inserted into the pBlueBac4.5/V5-His expression vector in frame with the C-terminal His.Tag and V5 epitope to give a theoretical protein of expected mass 42,679 Da under the transcriptional control of the baculovirus polyhedrin promoter. The nucleotide sequence of the insert was determined to be exactly the same as that submitted to GenBank under accession no. AK014204. Sf21 insect cells were co-transfected with the pBlueBac-Nudt13 DNA construct and Bac-N-Blue viral DNA and pure recombinant Nudt13 baculovirus isolated by plaque assay and purification.

**Fig. 1:**
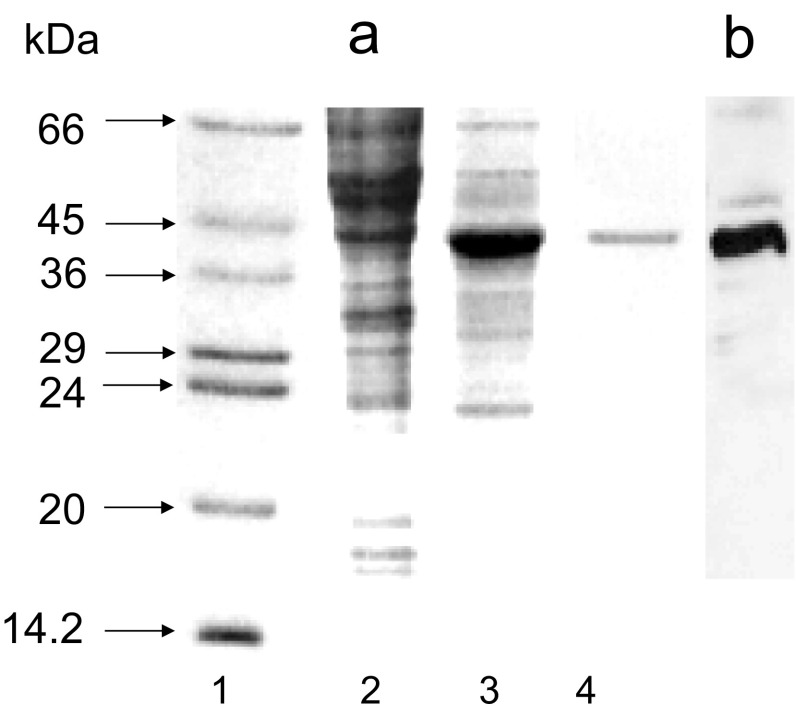
Expression of Nudt13 in High Five™ cells. High Five™ cells were infected with recombinant Nudt13 virus at a MOI of 10 for 48 h. Samples were analysed by SDS-PAGE (15% w/v) and stained with Coomassie Blue. **a** *Lane 1* protein standards: bovine serum albumin (66 kDa), ovalbumin (45 kDa), glyceraldehyde 3-phosphate dehydrogenase (36 kDa), carbonic anhydrase (29 kDa), trypsinogen (24 kDa), soybean trypsin inhibitor (20 kDa) and α-lactalbumin (14.2 kDa); *lane 2* control uninfected High Five™ cells; *lane 3* high Five™ cells infected with Nudt13 virus for 48 h; *lane 4* purified Nudt13. **b** Immunoblot analysis of Nudt13 (the same cells as in **a**, *lane 3*) using His.Tag monoclonal antibody

High Five insect cells were then infected with pure Nudt13 virus. SDS-PAGE analysis of a cell lysate 48 h after infection showed the presence of a major band corresponding to a 42 kDa protein in cells infected with Nudt13 virus which represented more than 50% of the total cell extract and which was not present in uninfected cells (Fig. 1a). The expression of Nudt13 was confirmed by western blotting using an anti-His.Tag monoclonal antibody which detected the C-terminal His.Tag of the recombinant Nudt13 (Fig. 1b). It was purified to homogeneity by affinity chromatography on NiCAM-HC resin (Fig. 1a, lane 4).

### Substrate Specificity and Reaction Requirements of Nudt13

Among the substrates tested, Nudt13 showed a high degree of specificity towards NADH and NADPH compared with other related nucleotides when assayed at a fixed concentration of 0.5 mM. Low activity was found with Ap_2_A, NAD^+^_,_ NADP^+^_,_ FAD and ADP-ribose and little or no activity with other nucleotides examined (Table 1). With NADH as substrate, Nudt13 displayed optimal activity at alkaline pH, between pH 7.8 and 8.2, with about 50% activity remaining at pH 7.0 and 9.0. The enzyme was absolutely dependent on a divalent metal cation for its activity, with 2–5 mM Mn^2+^ proving optimal for all substrates tested. The optimal Mg^2+^ concentration was unusually high at between 40 and 100 mM, giving about threefold lower activity than 2 mM Mn^2^; only 20% maximum activity remained at 5 mM Mg^2+^. The enzyme obeyed simple Michaelis–Menten kinetics with NADH as substrate in the presence of 2 mM Mn^2+^. *K*_m_ and *k*_cat_ values were determined for NADH under optimal assay conditions by non-linear regression analysis of data obtained by HPLC analysis and were 0.34 mM and 7 s^−1^.

**Table 1:** Substrate specificity of Nudt13 was determined colorimetrically at a fixed substrate concentration of 0.5 mM

Substrate	Relative activity (%)	Substrate	Relative activity (%)
NADH	100	Ap_4_A	2
NADPH	92	Ap_6_A	0.5
NAD^+^	7	UDP-glucose	2
NADP^+^	4	UDP-galactose	0.5
FAD	7	Canonical NTPs	<0.1
ADP-ribose	5	Canonical dNTPs	<0.1
Ap_2_A	14	8-oxo-dGTP	<0.1
Ap_3_A	4	5-Me-CTP	<0.1

The activity was expressed relative to NADH hydrolysis under the same conditions, where 100% was 5.7 µmol NADH hydrolyzed min^−1^ mg^−1^ protein. Values are the averages of duplicate determinations

### Product Analysis

**Fig. 2:**
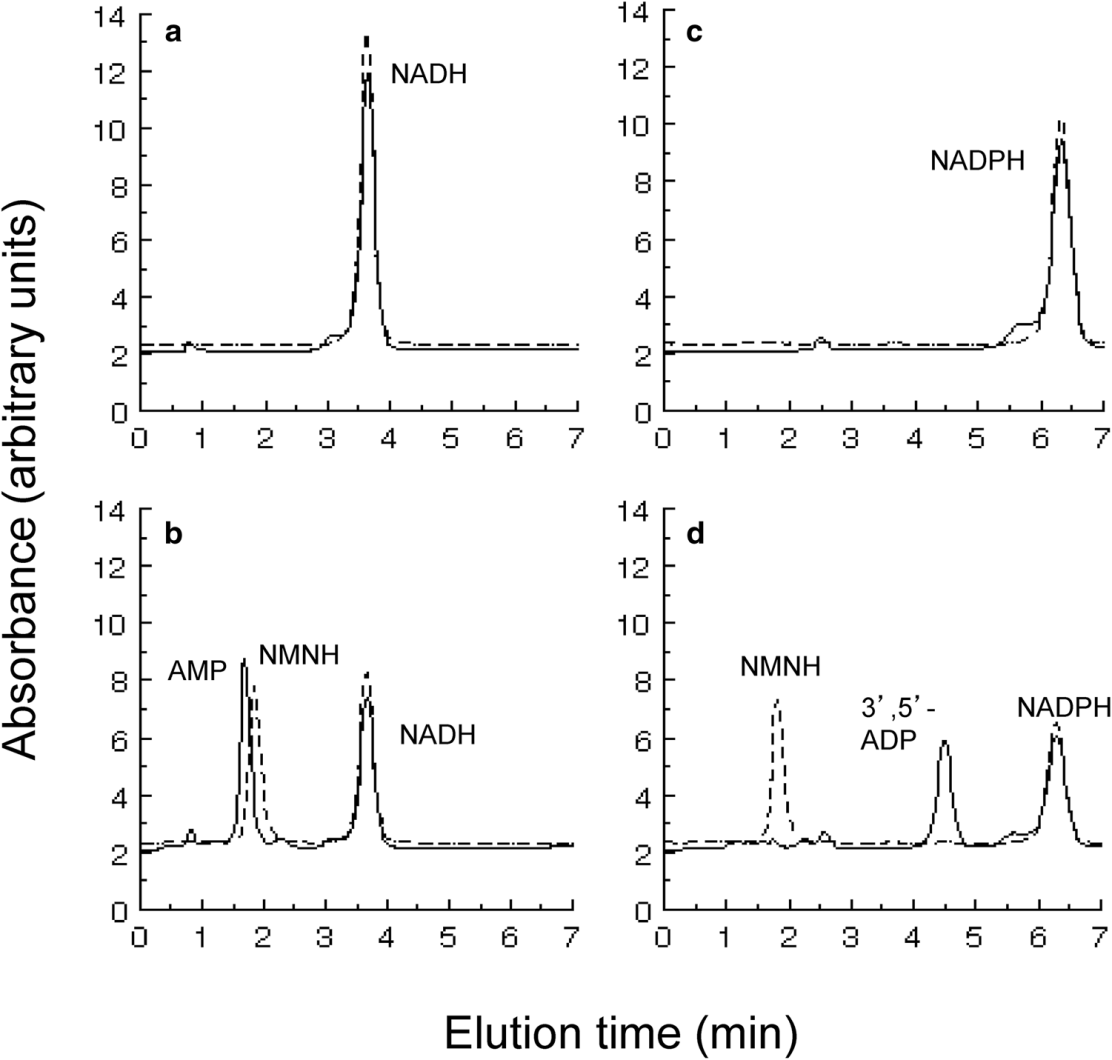
Determination of reaction products of NADH and NADPH hydrolysis by Nudt13. The reaction mixtures contained 0.25 mM NADH (**a, b**) or NADPH (**c, d**) and were incubated at 37 °C for 10 min without (**a, c**) or with (**b, d**) 0.25 µg Nudt13 and the products were separated by HPLC. Absorbance at 259 nm (*lines*); absorbance at 340 nm (*dashed lines*). Products were identified by comparison to authenticated standards

To determine the products of NADH and NADPH hydrolysis by Nudt13, aliquots of reaction mixtures containing each substrate were analysed by HPLC. The disappearance of substrate was accompanied by the appearance of AMP and NMNH in the case of NADH (Fig. 2a), and 3′,5′-ADP and NMNH in the case of NADPH (Fig. 2b).

### Subcellular Localization of Nudt13

**Fig. 3:**
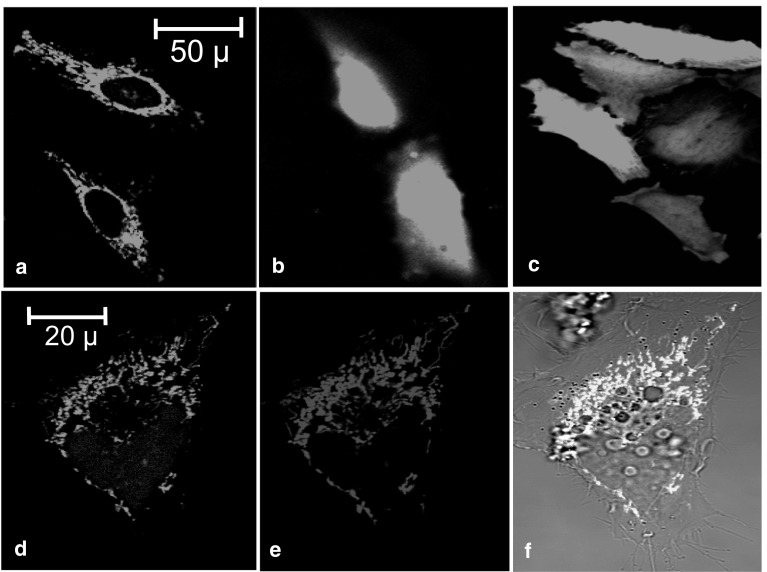
Subcellular localization of Nudt13 by fluorescence confocal microscopy. **a** EGFP fluorescence of HeLa cells transfected with pNudt13-EGFP; **b** EGFP fluorescence of HeLa cells transfected with pEGFP-Nudt13; **c** EGFP fluorescence of HeLa cells transfected with the pEGFP-C2 vector; **d** EGFP fluorescence of a sample cell transfected with pNudt13-EGFP; **e** red fluorescence of the same cell in **d** stained with MitoTracker red CM-H_2_XRos; **f** superimposition of **d** and **e** on the bright field picture of the same cell

The subcellular localization of Nudt13 was determined by expression of the protein in HeLa cells as N- and C-terminal fusions with EGFP. HeLa cells transfected with pNudt13-EGFP showed a distinctive pattern of fluorescence characteristic of mitochondria (Fig. 3a), while cells transfected with pEGFP-Nudt13, in which the putative N-terminal mitochondrial targetting signal is masked, showed a diffuse nucleo-cytoplasmic fluorescence (Fig. 3b) similar to EGFP alone (Fig. 3c). The mitochondrial localization of Nudt13 in cells transfected with pNudt13-EGFP (Fig. 3d) was confirmed with Mitotracker Red CM-H_2_XRos staining (Fig. 3e). Superimposition of both green and red fluorescence resulted in a yellow image with both signals clearly coincident (Fig. 3f).

## Discussion

Eukaryotic members of the Nudix hydrolase subfamily possessing the “SQPWPFPxS” sequence motif characterized so far are known or predicted to be peroxisomal—*Saccharomyces cerevisiae* NPY1 [3, 8], *Caenorhabditis elegans* ndx-9 [3], *Homo sapiens* NUDT12 [6] and *A. thaliana* AtNUDX19 [14], with AtNUDX19 having a dual chloroplastic location [11]. Such locations are not surprising as many reactions in these organelles are dependent upon reduced pyridine nucleotide cofactors. Another subcellular compartment where such activities would be expected is the mitochondrion. In rat hepatocytes, free mitochondrial NADH has been measured at 300–400 µM and NADPH at 4 mM while the corresponding figures for NAD^+^ and NADP^+^ are 4–6 and 1 mM respectively [15]. Compared to other Nudix NADH pyrophosphohydrolases characterized so far, Nudt13 exhibits a strong substrate preference for NAD(P)H over any other substrates tested and so a role for Nudt13 and its human ortholog NUDT13 in the regulation of NAD(P)H pools can be suggested. Nudt13 might also serve to generate NMNH, which may have a specific function within the mitochondrion [16].

Nudt13 has the same domain architecture as *A. thaliana* AtNUDX19. The latter enzyme has a marked preference for NADPH over NADH [11] and analysis of pyridine nucleotide levels in *nudx19* deletion mutants has shown an increase in intracellular NADPH, but not NADH [17]. This along with other phenotypic features of the mutant cell lines has suggested that AtNUDX19 is a key factor in the regulation of NADPH pools and redox control in this organism [17, 18]. Nudt13 does not display the same preference for NADPH in vitro as AtNUDX19; however, a more detailed analysis of substrate utilization in vitro than that presented here is unlikely to reveal the true substrate profile and preference of the mammalian NUDT13 subfamily in vivo, given the known difficulties in inferring these from in vitro activities [2, 19]. A good illustration of this is the unusual divalent ion requirement of Nudt13. Optimal activity in vitro was obtained in the presence of 2–5 mM Mn^2+^or 40–100 mM Mg^2+^, both of which are highly unphysiological. Free matrix Mg^2+^ has been measured as 0.67 mM [20, 21] while free Mn^2+^ is unlikely to be greater than 1 µM [22]. The activity of Nudt13 measured at 0.67 mM Mg^2+^ was only about 1% of the maximum observed and was negligible at 1 µM Mn^2+^. The microenvironment of Nudt13 within the mitochondrial matrix may of course alter the divalent ion requirement to match the physiological setting. Alternatively, substrates as yet untested may prove to have low *K*_m_ values and divalent ion optima. By analogy with the MutT and NUDT1 (MTH1) 8-oxo-dGTPases [23, 24], these could include ring-oxidized or other non-functional metabolites of the pyridine nucleotides [25] with hydrolysis removing them from the functional pyridine nucleotide pools to prevent toxicity. Another possibility arises from the finding that the *E. coli* NudC NADH pyrophosphohydrolase [26] removes NMN from NAD^+^-capped small regulatory RNAs much more efficiently than it hydrolyzes NADH [27, 28]. Regulatory micro-RNAs have been detected in mitochondria [29] but there is currently no evidence that they are capped by NAD^+^. Thus, although a role for Nudt13 in mitochondrial pyridine nucleotide metabolism seems the most likely by analogy with AtNUDX19, a true understanding will require a full phenotypic analysis of a deletion mutant, including measurements of pyridine nucleotide levels.

Assuming that mitochondrial NADH and/or NADPH are the relevant substrates for Nudt13, what might its role be? The NAD(P)^+^/NAD(P)H ratios are important regulators of the redox state of the cell and of numerous enzymic activities and signalling processes and may act as redox sensors for transcriptional control [30–32]. In particular, mitochondrial NADPH is required for the reduction of oxidised glutathione and for thioredoxin regeneration while NADH can be used for the generation of reactive oxygen species from the electron transport chain. How cellular responses to oxidative stress might be affected by Nudt13 activity will depend on how it is regulated in response to physiological signals. Induction or activation would favor NAD(P)H hydrolysis and an increase in NAD(P)^+^/NAD(P)H ratios while repression or inhibition would have the opposite effect. Such a ratio change could occur independently of redox reactions and could be a transient reponse as NADH at least can be regenerated from NMNH and ATP by the mitochondrial enzyme NMNAT3 [16]. Its influence could also extend to the cytosol as result of the NADH and NADPH shuttles that can transfer reducing equivalents across the mitochondrial membrane [32, 33]. That the human *NUDT13* gene is subject to regulation has been shown by the direct correlation of its expression with that of the proposed tumor suppressors MFSD4 and occludin (OCLN) and the inverse correlation with that of the metastasis-promoting bone morphogenetic protein 2 (BMP2) in several gastric cancer cell lines [33]. Increased OCLN and decreased BMP2 expression inhibit the epithelial-mesenchymal transition (EMT), an important stage in tumor cell invasion of tissues. This study suggests that both the uncharacterized MFSD4 and NUDT13 may have a role in the regulation of the EMT. Increased NADPH oxidase activity has been associated with induction of the EMT [34, 35] so it would be interesting to establish whether up-regulation of NUDT13 can reduce the supply of cytosolic NADPH.

Other Nudix hydrolases known to be located in mammalian mitochondria are the NUDT9 ADP-ribose hydrolase [36, 37] and a portion of the NUDT1 (MTH1) 8-oxo-dGTPase [38] while *Arabidopsis* has confirmed mitochondrial Nudix hydrolases that are active towards coenzyme A derivatives (AtNUDX15) and long-chain diadenosine polyphosphates (AtNUDX13) [11, 39]. Recent studies have focussed on the possible role of NUDT9 and the cytosolic NUDT5 in the catabolism of mitochondrial NAD^+^ and its metabolites [40–42] while many other studies have addressed the dynamic regulation of pyridine nucleotides and energy homeostasis in this organelle [43, 44]. The essential role of NAD(P)^+^ and nudix proteins in DNA damage repair, ageing and neurodegeneration linked to mitochondrial homeostasis is now also well recognized [45, 46]. However, none of these studies has considered the possible role of Nudt13 in these processes, most probably because details of its activity are not present in the primary literature. Thus, the simple characterization presented here should now serve to draw attention to this protein and lead to its consideration in future analyses of pyridine nucleotide metabolism and function in the mitochondria and other cellular compartments.

## Abbreviations

Ap_2_A: Diadenosine 5′,5‴-*P* ^1^,*P* ^2^-diphosphate (other diadenosine polyphosphates are abbreviated similarly)
EGFP: Enhanced green fluorescent protein
MOI: Multiplicity of infection
